# Modelling taxi drop-off decisions in FIFO lanes via survival and discrete choice analysis

**DOI:** 10.1371/journal.pone.0331664

**Published:** 2025-09-08

**Authors:** Fangyi Yang

**Affiliations:** 1 College of Intelligent Science and Control Engineering, Jinling Institute of Technology, Nanjing, China; 2 Jiangsu Key Laboratory of Intelligent and Low-Carbon Transportation, Nanjing, China; 3 School of Mechanical Engineering, Nanjing University of Science and Technology, Nanjing, China; Chang'an University, CHINA

## Abstract

Traffic congestion frequently occurs in the drop-off zones of large integrated passenger hubs, posing significant challenges to the efficient utilization of lane space. This study develops a First-In-First-Out (FIFO) taxi drop-off decision-making model, incorporating both static and dynamic Logit frameworks grounded in panel data analysis. The model accounts for heterogeneity across vehicles, temporal variations, and spatial factors influencing drop-off decisions. Key explanatory variables include the probability of current forced parking time reaching the ‘waiting patience’ threshold, the relative parking position of the vehicle, the likelihood of the current parking spot being the expected one, and the ratio of travel time. A mixed distribution model of passenger patience was constructed through survival analysis and calibrated to reflect empirical data accurately. The model estimation results indicate that passenger patience significantly influences drop-off decisions. All models—static, dynamic, and Cox proportional hazards—achieved prediction accuracies exceeding 70%, with the dynamic model outperforming others when ample sample data is available. This study is novel in integrating both panel data-based discrete choice models and survival analysis to describe dynamic taxi drop-off decisions in congested FIFO lanes. The behavioral insight into the sunk cost effect – where drivers tend to wait longer after repeated forced stops – is quantitatively confirmed. Furthermore, the model introduces interpretable explanatory variables that reflect real-world operational constraints and driver psychology. To further elucidate the temporal dynamics of drop-off behavior, the study integrates survival analysis, particularly the Cox proportional hazards model. Findings reveal that extended forced parking durations, longer travel times, and increased instances of forced stops are associated with decreased hazard rates, suggesting a higher tolerance for prolonged waiting. This behavior is attributed to the sunk cost effect, where drivers, having invested time in waiting, are more inclined to continue waiting rather than proceed to drop off passengers. These insights underscore the necessity of incorporating driver psychological behaviors into traffic management strategies. Recommendations include implementing ‘no-waiting zones’ in drop-off areas, establishing mandatory drop-off time thresholds based on median survival times, introducing parking pricing policies for prolonged stops to internalize spatial costs, and utilizing real-time guidance systems (e.g., variable message signs) to prompt timely passenger drop-offs. Such measures aim to mitigate the adverse effects of prolonged forced stops and enhance the operational efficiency of drop-off zones.

## Introduction

At integrated passenger transport hubs (such as airports and high-speed rail stations), passengers arrive at designated drop-off areas via buses, taxis, private cars, and other modes before entering the waiting hall. However, in the “first-in, first-out” (FIFO) drop-off lane, vehicles must stop sequentially and wait for drop-off. This single-lane FIFO operation mode is quite common at China’s high-speed railway stations—for example, at Nanjing South Railway Station, Zhengzhou East Railway Station, Beijing South Railway Station, and Guangzhou South Railway Station. Such an operation may lead to inefficient utilization of available parking space, which in turn exacerbates congestion in the drop-off area.

Several studies have addressed similar congestion issues in drop-off lanes. Zhang et al. [1] empirically analyzed passenger drop-off behaviors at Guangxi Airport and identified behavioral complexity as a key determinant of dwell time. Dong and Ryerson [2] explored the impact of ride-hailing services on airport taxi trips and parking turnover, revealing reduced taxi usage and modest effects on parking volume post-implementation of UberX and Lyft. Yang et al. [3] investigated congestion mitigation strategies for FIFO drop-off lanes and demonstrated that removing batch-based entry control could significantly increase taxi throughput. Their simulation results further supported enforced drop-off strategies beyond expected locations to enhance efficiency. As an extension, Giro et al. [4] identified socio-demographic and trip attributes affecting service quality perception, reinforcing that ride-hailing services were perceived more favorably than traditional taxis. In a later study, they modeled the demand for taxis and ride-hailing transport services (RTS) in the Jakarta Greater Area using both dynamic and demand-supply models, highlighting superior performance of RTS in terms of cost and time savings [5]. These studies highlight the importance of both behavioral insight and operational policy in enhancing drop-off area performance.

Meanwhile, parking management strategies, particularly those involving dynamic pricing, have also been shown to influence space utilization and reduce dwell time. Magsino et al. [6] assessed time- and space-based parking fee schemes within Smart Parking Management Systems (SPMS), offering empirical evidence to support socially optimal pricing adjustments. Qin et al. [7] proposed a dynamic pricing strategy for street parking in Beijing’s commercial zones, demonstrating that parking occupancy rates could be balanced, and illegal parking reduced by adjusting rates based on demand.

This study aims to integrate survival analysis with discrete choice models to investigate forced parking behavior from two complementary perspectives: event dynamics and decision mechanisms. Specifically, survival analysis methods are employed to explore the factors influencing the duration of forced parking, while discrete choice models are used to analyze drivers’ decision-making processes during parking. The ultimate objective is to provide both a solid theoretical foundation and practical guidance for optimizing drop-off area management and enhancing parking space utilization.

Discrete choice models, particularly Logit-based frameworks, have been extensively used in transportation studies to model individual preferences across a wide range of behavioral scenarios. Based on their focus, existing works can be broadly categorized into three groups: studies exploring individual preferences in travel behavior, research innovating discrete choice modeling methodologies, and approaches integrating discrete choice with machine learning technique. Studies such as Yazici [8], Hasnine [9], Souza [10], and Guzman [11] delve into user preferences across airport taxi services, tour-based travel, freight mode selection, and fare evasion behavior, respectively. These works emphasize the behavioral heterogeneity underlying different transport contexts. Meanwhile, Antonini [12], Hassan [13], Rambha [14], and Urata [15] have advanced model specifications to address pedestrian movements, latent choice sets, evacuation behavior, and idle driver repositioning. Lastly, Wang [16], Yao [17], and Nimale [18] introduced novel integrations of deep neural networks, discrete choice modeling with clustering algorithms, and copula-based joint modeling to improve prediction and interpretability. These studies collectively demonstrate the adaptability of discrete choice models across varied applications. However, few have addressed the temporal evolution of drop-off decisions in FIFO settings, where past experiences influence current choices-a gap this study aims to fill using dynamic Logit modeling.

With the increasing availability of panel data sets and rapid advancements in statistical and econometric models, nonlinear models for estimating binary panel data have garnered considerable attention. Panel data structures allow for the inclusion of individual-specific intercepts and state-dependent lagged response variables—key features that capture so-called state dependence, i.e., how an experience at one moment affects the likelihood of experiencing the same event in the future [19]. The dynamic Logit model [20] offers an intriguing method for dynamically analyzing binary panel data. However, because of the inherent parameter problems [21,22], maximum likelihood (ML) estimates of parameters in binary fixed-effects panel models are inconsistent. Two primary approaches have been proposed to address these methodological challenges: the first reduces the bias order of the ML estimator to $O\left(T^{-2}\right)$ by correcting the parameter estimator deviation using modified likelihood functions [23–26] or scoring functions [27–30], while the second employs conditional reasoning [31,32] with conditional maximum likelihood (CML) estimators that remain consistent when the time series *T* is fixed. Both approaches can mitigate the incidental parameter problem. CML estimation has been widely implemented in static Logit models [33–35] and quadratic exponential (QE) models [36,37]. For dynamic Logit (DL) models, however, obtaining sufficient statistical data for a single intercept is impractical; hence, Honore & Kyriazidou (2000) [38] derived the weighted CML estimator, and Bartolucci & Nigro (2012) [39] later proposed a pseudo-CML (PCML) estimator based on an approximate QE model. When *T* is large, the computational burden of CML estimation can become excessive, prompting Bartolucci to propose a general recursive algorithm that efficiently calculates the conditional likelihood for some binary data models [40]. This development enhances the practicality of dynamic Logit models in transportation applications.

Survival analysis, primarily used for handling time-to-event data, is highly effective in extracting the dynamic characteristics of events. In transportation research, survival analysis has been applied to predict vehicle delay times and passenger waiting times, among other phenomena. For example, the Kaplan–Meier estimator has been used to plot survival curves that reveal how various factors influence vehicle delays and pedestrian crossing waiting times [41–43], while the Cox proportional hazards model has been applied to assess the impact of road conditions, traffic volume, and other factors on vehicle delay risks [44]. While survival analysis focuses on the temporal aspects of events, discrete choice models capture the process by which individuals select among multiple alternatives. Combining these two methodologies enables a comprehensive understanding of the dynamic decision mechanisms underlying traffic behavior. For instance, a driver’s decision when forced to wait for a parking space (as analyzed through discrete choice modeling) may be influenced by the actual waiting duration (as captured through survival analysis); conversely, the length of the waiting period itself might be affected by the driver’s drop-off strategy. Thus, integrating survival analysis with discrete choice models provides deep insights into both the time-dynamic and decision-making aspects of drop-off behavior, offering new methods and perspectives to address congestion in drop-off areas.

This study contributes to the literature in three significant ways: (1) It combines survival analysis with discrete choice models to simultaneously model both the timing and decision-making process of taxi drop-offs, which has been rarely addressed together in prior research. (2) A unique panel dataset of taxi trajectories at a major high-speed rail terminal is constructed and analyzed using dynamic Logit and Cox regression models, providing practical insights for real-word application. (3) Practical policy implications are proposed based on survival time thresholds and dynamic risk indicators, such as the implementation of “no-wait zones”, drop-off time thresholds, VMS(variable message signs)-based real-time drop-off guidance, and a time-based parking fee scheme to limit taxi waiting times and encourage efficient turnover.

The remainder of this paper is structured as follows: Section 2 describes the data sources and preprocessing steps. Section 3 presents the modeling framework, including both survival analysis and discrete choice models. Section 4 reports the empirical results and compares model performances. Section 5 concludes the study by integrating the policy implications and outlining future research directions.

## Data and methods

The taxi drop-off data for the integrated hub is primarily obtained from real-time monitoring systems. Cameras continuously record key information including parking locations, drop-off times, and vehicle queue lengths. Within the taxi drop-off lane, vehicles can be in one of three states: moving, waiting, or dropping off. Specifically, when a downstream vehicle stops to drop off, it forces the subsequent vehicle to transition from “moving” to “waiting.” The waiting vehicle then either resumes movement to secure an ideal parking position or, if its stopping time exceeds a predetermined threshold (i.e., waiting patience), it commits to dropping off its passengers (yang, 2020) [1].

**Fig 1:**
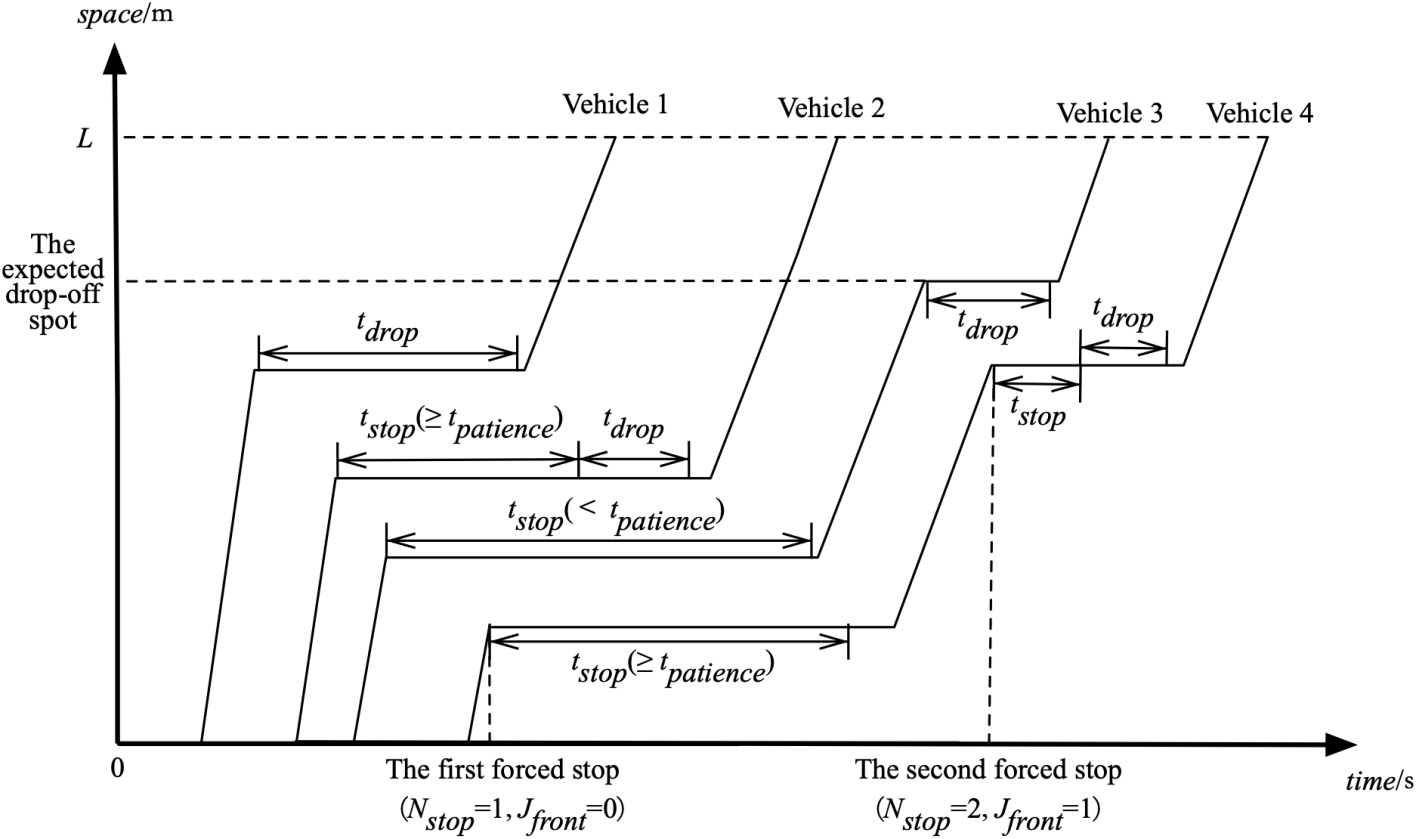
Spatio-Temporal Diagram of Vehicle Trajectory in FIFO System.

To better illustrate the data and modeling framework, a spatiotemporal trajectory diagram of vehicles operating under a FIFO (First-In, First-Out) drop-off system is shown in Fig 1. The figure presents four vehicles and their sequential behaviors within the lane. Vehicle 1, acting as the leading vehicle, stops exactly at its expected drop-off position and completes the drop-off with a duration denoted as $t_{drop}$ (drop-off time). Vehicle 2 follows Vehicle 1 and also drops off, but only after a period of forced stop, with a stopping duration $t_{stop}$ that is greater than or equal to the passenger’s waiting patience, denoted as $t_{patience}$ (i.e., $t_{stop}\geq t_{patience}$). In contrast, Vehicle 3 does not drop off during its first forced stop because its forced stop duration is less than the patience threshold ($t_{stop}<t_{patience}$), and instead it proceeds to drop off at its expected parking position. Vehicle 4 experiences two forced stops: it does not drop off after the first stop (forced stop count $N_{stop}=1$, and the front vehicle did not drop off, $J_{front}=0$), but does drop off after the second forced stop ($N_{stop}=2$, $J_{front}=1$). This figure helps clarify how different vehicles respond under varying forced stop conditions, and it visually supports the explanation of key variables used in the survival and discrete choice modeling process.

To ensure the robustness of our conclusions, we analyzed taxi trajectory data from the drop-off lanes at Nanjing South Railway Station during two different time periods: a morning session (Period 1) and an afternoon session (Period 2). Each dataset corresponds to approximately one hour of continuous observation. In Period 1, a total of 487 taxis were recorded to have completed a drop-off in the FIFO lane, of which 444 underwent at least one forced stop behind another vehicle. Similarly, in Period 2, 403 taxis were observed, with 362 experiencing forced stops. For both periods, the first forced stop events totaled 444 (Period 1) and 362 (Period 2), while second to fourth forced stop events accounted for an additional 39 and 34 instances, respectively. These datasets provide a representative view of taxi behaviors under typical operational conditions and support comparative analysis between different time windows.

### Variable definition

To comprehensively capture taxi drop-off behavior, the analysis distinguishes between variables suited for survival analysis and those used in the discrete choice model. Each set of variables serves a specific modeling purpose, reflecting different aspects of driver and passenger behavior. This classification helps improve estimation accuracy and interpretability across different empirical methods.

#### 1 ) Survival analysis variables.

These variables are selected to analyze the time-to-event (forced stop duration) nature of the drop-off process. By focusing on temporal characteristics, survival analysis captures the dynamics of waiting behavior under different influencing factors. This allows for a better understanding of how long a taxi will wait before deciding to drop off passengers.

(1) Forced Stop Duration (Survival Time, $t_{stop}$)

For vehicles that are required to drop off passengers, the forced parking duration can be categorized into two types:

**Forced Stop Duration Leading to Drop-off**: In this case, the duration of the forced stop exceeds the driver’s waiting tolerance. This excess indicates that the driver has waited long enough—i.e., beyond his/her predetermined waiting patience—to trigger a drop-off decision. The measured forced stop time for these vehicles reflects the waiting tolerance. This provides uncensored observations that are critical for fitting patience distributions.

**Forced Stop Duration Not Leading to Drop-off**: Here, the vehicle’s forced stop duration is less than its waiting tolerance. As a result, the taxi does not proceed with the drop-off, and the recorded “stop time” only represents the period of forced parking, with the actual waiting tolerance remaining unobserved. Such data are treated as right-censored in survival models and contribute to estimating the hazard function.

(2) Drop-off Behavior (Outcome Event, $y_{i}$)

A binary indicator where a value of 1 indicates that the vehicle drops off, and 0 indicates that it does not. This variable captures the outcome event of interest in survival analysis—i.e., whether the observed parking duration results in a drop-off. It serves as the dependent variable in the Kaplan-Meier and Cox regression models, enabling estimation of survival probabilities and hazard functions under different covariates.

(3) Categorical Variables

These include:

- The number of forced stops ($N_{stop}$): This indicates how many times a vehicle has been forced to stop without successfully dropping off passengers.
- Whether the vehicle in front has dropped off ($J_{front}$): This variable reflects the potential influence of queue behavior, where drivers may imitate the actions of preceding taxis.

These categorical variables are used to stratify survival curves, allowing researchers to assess how behavior patterns change under different conditions. For example, more forced stops may indicate increasing frustration, while the status of the preceding vehicle may serve as a behavioral cue.

(4) Continuous Variables

These include:

- Cumulative forced stop duration ($t_{cum}$: t the sum of all previous forced stop durations prior to the current one, capturing historical waiting cost.
- Drop-off time ($t_{drop}$): an indicator of passenger-specific unloading requirements, often influenced by luggage or number of passengers.
- Vehicle location ($L_{i}$): the spatial position of the vehicle within the FIFO lane.
- Travel time ($t_{travel}$): the total travel time of the vehicle from entry into the taxi system to the current point.
- Drop-off probability of the current location ($P_{loc}$): estimated from spatial patterns of drop-off events based on historical data.
- Drop-off probability of the current forced stop duration ($P_{patience}$): calculated using the hybrid distribution model of waiting patience.

Together, these variables form the backbone of the hazard-based and choice models by quantifying both historical and real-time features of the driver’s operational context. Their inclusion allows for nuanced behavioral modeling, aligning with both statistical rigor and observed field dynamics. The drop-off probability of the current location and the drop-off probability of the current forced stop duration in the above variables are explained.

**Fig 2:**
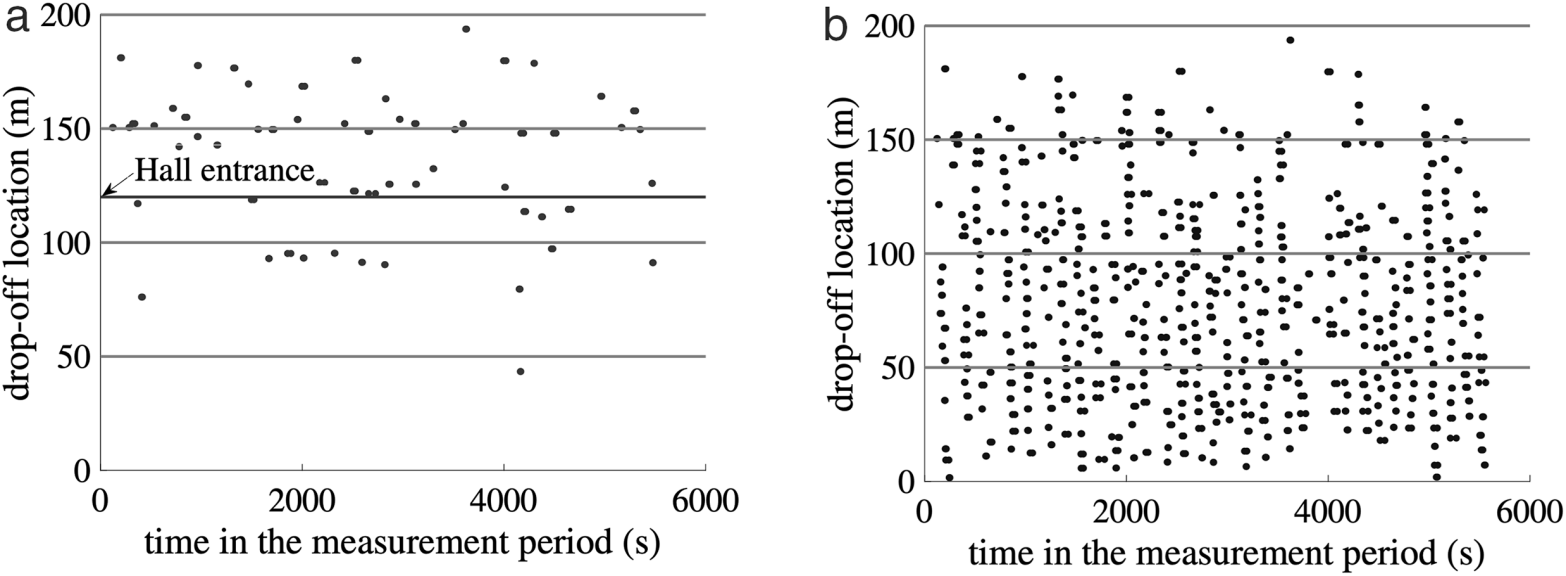
The Drop-off Locations of Taxis for a Morning Peak Period: (a) Head taxis; (b) All taxis.

The drop-off probability of the current location is derived by analyzing the spatial distribution of drop-off positions in a congested FIFO lane. This is based on empirical trajectory data collected at Nanjing South Railway Station, which shows that head taxis tend to drop off near active areas (e.g., the entrance or ticket hall), resulting in a higher proportion of drop-offs near the entrance (see Fig 2). Most head taxis stopped between the hall entrance (120m) and the ticketing service (170m) to facilitate passengers’ access to the hall; see Fig 2a for the drop-off locations of head taxis for a morning peak period of about 1.5hours measured on the site. The lane space between 160-200m was thus very underutilized; see Fig 2b for the drop-off locations of all the taxis for the same peak period.

Similarly, the drop-off probability for the current forced stop duration is modeled using a hybrid Gamma distribution. This is designed to capture the heterogeneity in vehicles’ waiting tolerance—defined as the maximum time a driver is willing to wait before deciding to drop off. Based on the dataset, we identify two types of observations: (1) vehicles that drop off passengers at a forced stop (uncensored data), and (2) vehicles that do not drop off (right-censored data). To properly model both types, we adopt a mixture model combining two Gamma distributions with parameters ($k_{1}$, $\theta_{2}$) and ($k_{2}$, $\theta_{2}$), weighted by α and (1−α), respectively. These parameters are estimated from empirical data. The mixed probability density function is expressed as:


$$f\left(x;\alpha ,k_{1},\theta_{1},k_{2},\theta_{2}\right)=\alpha \cdot \frac{x^{k_{1}-1}e^{-\frac{x}{\theta_{1}}}}{\theta_{1}^{k_{1}}\Gamma \left(k_{1}\right)}+(1-\alpha )\cdot \frac{x^{k_{2}-1}e^{-\frac{x}{\theta_{2}}}}{\theta_{2}^{k_{2}}\Gamma \left(k_{2}\right)}$$ (1)

This model is estimated via maximum likelihood to derive the distribution of wait patience.

**Fig 3:**
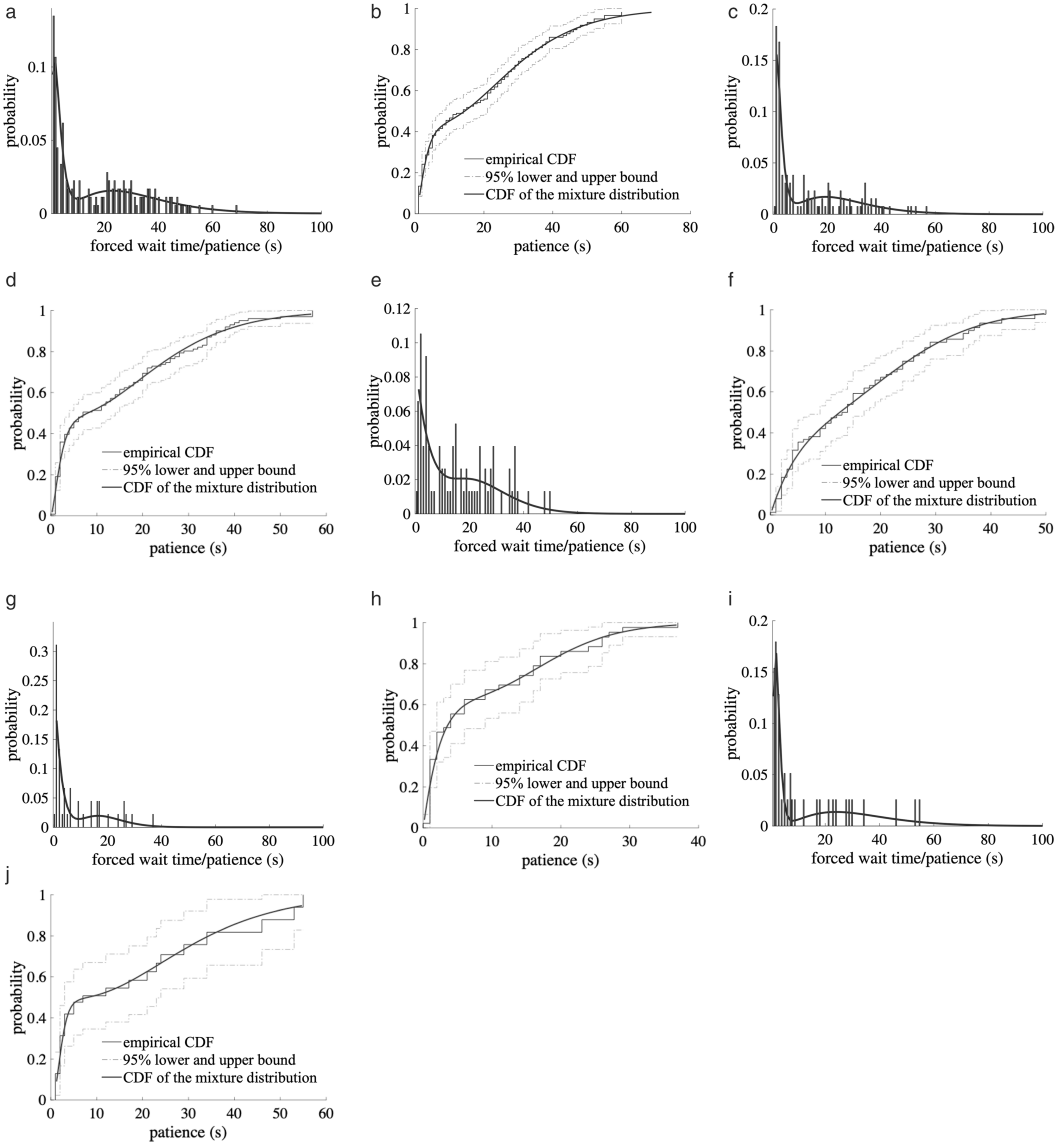
Distributions of Passenger Patience for the Period 1: (a) Histogram of Taxi Wait Times and PDF of Patience for 1^st^ Forced Stops in Zone 1; (b) CDFs of Taxi Wait Times and Patience for 1^st^ Forced Stops in Zone 1; (c) Histogram of Taxi Wait Times and PDF of Patience for 1^st^ Forced Stops in Zone 2; (d) CDFs of Taxi Wait Times and Patience for 1^st^ Forced Stops in Zone 2; (e) Histogram of Taxi Wait Times and PDF of Patience for 1^st^ Forced Stops in Zone 3; (f) CDFs of Taxi Wait Times and Patience for 1^st^ Forced Stops in Zone 3; (g) Histogram of Taxi Wait Times and PDF of Patience for 1^st^ Forced Stops in Zone 4; (h) CDFs of Taxi Wait Times and Patience for 1^st^ Forced Stops in Zone 4; (i) Histogram of Taxi Wait Times and PDF of Patience for 2^nd^-4^th^ Forced Stops in All Zones; (j) CDFs of Taxi Wait Times and Patience for 2^nd^-4^th^ Forced Stops in All Zones.

The curves of Fig 3(a), 3(c), 3(e), 3(g), and 3(i) display the probability density functions (PDFs) of the hybrid distribution for the five data types in the first time period. Specifically, subfigures (a) through (g) correspond to the first forced stops in Zones 1 through 4, respectively, while subfigure (i) presents the distribution for second to fourth forced stops across all zones. The overlaid histograms show the observed duration of forced taxi stops, and the smooth solid lines represent the fitted hybrid PDFs. Meanwhile, the smooth curves in Fig 3(b), 3(d), 3(f), 3(h), and 3(j) represent the cumulative distribution functions (CDFs) of the hybrid wait tolerance distribution. These CDFs are compared with the empirical CDFs derived as a supplementary outputs of the Kaplan-Meier estimator [45], used to approximate the survival function. Additionally, the Greenwood formula [46] was used to calculate 95% confidence intervals (upper and lower bounds). The hybrid CDFs show good agreement with the empirical CDFs and remain well within the 95% confidence range. Similar levels of fit were observed for both time periods analyzed.

The probability density function provides the basis for estimating how likely a vehicle is to reach its waiting threshold at any given moment during a forced stop. By referencing this distribution, the model can infer the real-time likelihood that a drop-off decision will be made, making it a critical input for both survival analysis and discrete choice modelling.

#### 2) Discrete choice model variables.

The discrete choice approach, particularly for dynamic Logit modeling, uses panel data to capture the sequential decision-making process regarding drop-off. The data structure is two-dimensional (time series and cross-section), which allows for the inclusion of individual-specific effects. The key components are:

- Explained Variable

Based on vehicle space-time trajectory data, each taxi’s drop-off decision is recorded as a binary outcome (1 if the taxi drops off at a forced stop, 0 otherwise). For example, the first forced stop data set comprises 444 taxis with stopping times ranging between 1 and 64 seconds. These panels are unbalanced; some taxis have as many as 64 observations while others have as few as 2.

- Explanatory Variables

Derived from survival analysis outputs, these include variables that capture temporal, spatial, and behavioral characteristics. Specifically:

- $X_{1}$: The probability that the current forced stop duration t is equivalent to wait patience, defined as (τ: the cumulative distribution function (CDF) of waiting patience τ, representing the probability that a driver’s patience threshold is less than or equal to the current stop duration. F(t): the cumulative distribution function (CDF) of waiting patience τ. f(t): the probability density function (PDF) of τ):

$$X_{1}=P\left\{0\leq \tau \leq t\right\}=F(t)=\int_{0}^{t}f(t)dt$$ (2)

- $X_{2}$: Vehicle relative parking position, calculated as (l: the current position of the vehicle in the FIFO lane. L: the total length of the FIFO lane used for passenger drop-off):

$$X_{2}=\frac{l}{L}$$ (3)

- $X_{3}$: The probability that the current parking position is the expected drop-off spot. It is defined via the nonparametric distribution (ι: a random variable representing the location of actual drop-off events. $l_{k}$: Discrete positions along the lane where observed drop-offs occur. $P\left\{\iota =l_{k}\right\}$: The empirical probability that a drop-off occurs at position $l_{k}$):

$$\begin{array}{c} X_{3}=P\left\{0\leq \iota \leq l\right\}=F(l)=\sum {\mathrm{\backslash nolimits}}_{l_{k}\leq l}P\left\{\iota =l_{k}\right\} \end{array}$$ (4)

- $X_{4}$: Driving time ratio, defined as the ratio of time spent since entering the drop-off area relative to total travel time $t_{a}$ ($t_{c}$: the time elapsed before the taxi entered the drop-off area):

$$X_{4}=\frac{t_{a}-t_{c}}{t_{a}}$$ (5)

- $X_{5}$: Forced parking count, which is considered only when a taxi has experienced more than one forced stop.

These explanatory variables enable dynamic modeling of decision-making behavior by incorporating past experiences and real-time positional data. The inclusion of both probabilistic and structural indicators ensures that the model is robust and interpretable.

### Data Pre-processing

To ensure model robustness and improve data quality, careful data preprocessing was conducted before estimation for both survival analysis and discrete choice models. This step is essential for addressing noise, handling data irregularities, and preparing the datasets for different modeling purposes.

#### 1) Survival analysis model data processing.

Extreme outliers in forced stop duration are eliminated to ensure robust estimates. Variables excluding survival time, final event indicators, and categorical variables are subjected to clustering analysis. A k-means clustering method is used to generate 2–3 class labels for key continuous variables (e.g., cumulative forced stop duration, drop-off time, vehicle location, travel time, and drop-off probability). These clusters enable distinct survival curve comparisons across different behavioral patterns.

#### 2) Panel data balancing for discrete choice models.

Since the panel data are inherently unbalanced (with varying numbers of forced stop observations per taxi), methods are applied to balance the data without discarding observations. This is achieved by supplementing missing sections through reasonable assumptions. For instance:

(1) When a Taxi Stops and Drops Off:

If a taxi’s stopping time t is less than or equal to the maximum forced stop duration $T_{m}$, the remaining data is supplemented so that the total stopping time equals $T_{m}$. It is assumed that all taxis drop off within this supplementary duration (coded as 1 in the explained variable).

(2) When a Taxi Is Forced to Stop but Does Not Drop Off:

If the stopping time is less than the waiting tolerance (with the final cumulative forced stop duration denoted by $\overset{―}{t_{c}}$), the explained variable may be coded as 0, indicating that the taxi has not dropped off. The supplementary rules ensure consistency in time series data completion without altering the original behavior.

## Model

### Survival analysis model

The survival analysis method is used to investigate the forced parking behaviour of taxis in the drop-off area, and a model is built to analyse the key factors affecting the duration of forced parking. Survival analysis not only focuses on whether an event occurs, but also emphasizes the duration of the event, which provides a powerful tool to reveal the dynamic characteristics of the vehicle waiting decision. The survival function and risk factors were modelled and analysed by Kaplan-Meier estimation, negative log-likelihood loss function, and Cox proportional risk model, respectively, using the forced stop duration as time-event data.

#### 1 ) Survival Function and Kaplan-Meier Estimation.

In survival analysis, the key object of interest is the survival function S(t), which represents the probability that the event of interest—in our case, the initiation of a drop-off—has not occurred by time t. Mathematically, the survival function is defined as


S(t)=P(T>t)=1−F(t) (6)

Where T denotes the forced stopping duration and F(t) is the cumulative distribution function. Since our analysis deals with time-to-event data, it is crucial to capture the evolution of the drop-off decision over time.

To nonparametrically estimate S(t), we employ the Kaplan–Meier estimator. This method is particularly well-suited for handling censored data (e.g., when a vehicle does not drop off within the observation period). The Kaplan–Meier estimate of the survival function is computed as:


$$\begin{array}{c} \hat{S}(t)=\prod {\mathrm{\backslash nolimits}}_{t_{i}\leq t}\left(1-\frac{d_{i}}{n_{i}}\right) \end{array}$$ (7)

where $t_{i}$ is a distinct time point at which a drop-off occurs, $d_{i}$ is the number of drop-off events at time $t_{i}$, and $n_{i}$ is the number of vehicles at risk just before $t_{i}$. This estimator allows us to graphically represent the drop-off “survival” (i.e., continuing the forced stop) over time and to compare survival curves across different subgroups (e.g., vehicles with different numbers of forced stops or situations with/without preceding drop-off events). Statistical tests such as the Log-rank test are used to assess the significance of differences between survival curves.

#### 2 ) Cox proportional hazards model and parameter estimation.

While the Kaplan–Meier method provides a descriptive view of the survival function, it does not quantify the influence of covariates on the timing of events. To this end, we employ the Cox proportional hazards model, which is a semi-parametric regression technique widely used for time-to-event data analysis.

The Cox model expresses the instantaneous hazard rate for experiencing a drop-off at time t, given a vector of covariates X, as:


$$h\left(t|X\right)=h_{0}(t)\text{exp}\left(\beta^{\text{T}}X\right)$$ (8)

Here, $h_{0}(t)$ is the baseline hazard function, β is a vector of regression coefficients, and $\text{exp}\left(\beta^{\text{T}}X\right)$ quantifies the relative risk. A key assumption of the Cox model is the proportionality of hazards: the ratio of the hazard rates for any two individuals remains constant over time.

Parameter estimation within the Cox framework is achieved via the partial likelihood method. For each observed event time $t_{i}$ at which a drop-off occurs (with event indicator $\delta_{i}=1$), the partial likelihood is given by:


$$L(\beta )=\prod_{i:\delta_{i}=1}\frac{\text{exp}\left(\beta^{\text{T}}X_{i}\right)}{\sum_{j\in R\left(t_{i}\right)}\text{exp}\left(\beta^{\text{T}}X_{j}\right)}$$ (9)

Where $X_{i}$ represents the covariates for the ith vehicle, and $R\left(t_{i}\right)$ is the risk set of vehicles still in the forced stop state immediately before $t_{i}$. The parameter vector β is then estimated by maximizing the logarithm of this likelihood function.

The negative log-partial likelihood (loss function) is expressed as:


$$\begin{array}{c} \text{Loss}(\beta )=-\sum {\mathrm{\backslash nolimits}}_{i:\delta_{i}=1}\left[\beta^{\text{T}}X_{i}-\text{ln}\left(\sum {\mathrm{\backslash nolimits}}_{j\in R\left(t_{i}\right)}\text{exp}\left(\beta^{\text{T}}X_{j}\right)\right)\right] \end{array}$$ (10)

Minimizing this loss function yields optimal estimates for β, and the exponential of these coefficients (i.e., exp(β)) provides the hazard ratios, which indicate the multiplicative change in the risk of drop-off associated with one-unit increments in the corresponding covariates.

This Cox model offers a robust framework for quantifying the effects of factors such as cumulative forced stop duration, vehicle position, travel time, and other relevant variables on the time until drop-off. Its use, in combination with the Kaplan–Meier estimator, allows for a comprehensive analysis of both the overall waiting dynamics and the relative risk contributions of individual factors in forced stopping scenarios.

### Discrete choice model

Because the data type is standard panel data, each observation is not independent. The same individual (vehicle) has multiple observation data, and there is a specific correlation between the observation data. Therefore, we can’t adopt the binary logit model, but the panel binary selection model can be adopted, including the static and dynamic logit models. We established the drop-off decision models for the first forced parking vehicle and the second or more forced parking vehicle.

#### 1 ) Static logit model.

For the sample of n taxis in the drop-off lane, each cab i (i=1,...,n) is observed at $T_{i}$ time points, allowing $T_{i}$ to vary between taxis to account for the unbalanced panel structure. Let the bivariate response variable of taxi i at time t be $y_{it}$ (explained variable), where $t=1,...,T_{i}$, $X_{i}$ is the column vector of the covariable (containing all explanatory variables), $X_{i}=\left(x_{i1},\ldots ,x_{iT_{i}}\right)$, and consider the static Logit formula based on the hypothesis:


$$p\left(y_{it}|\alpha_{i},X_{i}\right)=\frac{exp\left[y_{it}\left(\alpha_{i}+x_{it}^{T}\beta \right)\right]}{1+exp\left(\alpha_{i}+x_{it}^{T}\beta \right)}$$ (11)

Where $\alpha_{i}$ is the individual specific intercept, and vector β is the regression parameter related to the explanatory variable $x_{it}$. For the joint probability:


$$y_{i}={\left(y_{i1},\ldots ,y_{iT}\right)}^{T}$$ (12)

We can express it as:


$$p\left(y_{i}|\alpha_{i},X_{i}\right)=\frac{exp\left(\alpha_{i}y_{i+}\right)exp\left(\sum_{t}y_{it}x_{it}^{T}\beta \right)}{\prod_{t}\left[1+exp\left(\alpha_{i}+x_{it}^{T}\beta \right)\right]}$$ (13)

Among ∑t and ∏t scope for t=1,...,T and $y_{i+}=\sum_{t}y_{it}$ called total score.

#### 2 ) Dynamic logit model.

The dynamic model is a simple extension of the static Logit model, including $y_{i,t-1}$ in the set of covariables. In the dynamic Logit model, for a binary response sequence $y_{it}$, t=1,...,T, refers to the same vehicle *i*, corresponding to the covariable vector $x_{it}$. The conditional distribution of a single response is:


$$p\left(y_{it}|\alpha_{i},X_{i},y_{i0},\ldots ,y_{i,t-1}\right)=\frac{exp\left[y_{it}\left(\alpha_{i}+x_{it}^{T}\beta +y_{i,t-1}\gamma \right)\right]}{1+exp\left(\alpha_{i}+x_{it}^{T}\beta +y_{i,t-1}\gamma \right)}$$ (14)

γ is the regression coefficient of the lag response variable.

The inclusion of a single intercept $\alpha_{i}$ with unobstructed heterogeneity in a dynamic model creates what is known as the “initial condition” problem [47], which involves the correlation between time-invariant effects and the initial implementation $y_{i0}$. However, we treated individual unobserved effects as fixed parameters in the fixed effects method and gave the initial observations. The distribution of response vector $y_{i}$ with $y_{i0}$ as the condition is:


$$p\left(y_{i}|\alpha_{i},X_{i},y_{i0}\right)=\frac{exp\left(\alpha_{i}y_{i+}+\sum_{t}y_{it}x_{it}^{T}\beta +y_{i*}\gamma \right)}{\prod_{t}\left[1+exp\left(\alpha_{i}+x_{it}^{T}\beta +y_{i,t-1}\gamma \right)\right]}$$ (15)

The $y_{i*}=\sum_{t}y_{i,t-1}y_{it}$.

#### 3 ) Model estimation.

Unlike the static Logit model, the full DL model does not have sufficient statistics for the individual parameter $\alpha_{i}$. Therefore, conditional maximum likelihood (CML) inference is not feasible in the simple form. This paper adopts the pseudo-conditional maximum likelihood estimation and conditional likelihood function recursive calculation to estimate the dynamic model and improve the convergence rate of the estimation algorithm.

In this paper, we use a pseudo-CML estimator proposed by Bartolucci and Nigro (2012) [33] to estimate the parameters of the dynamic logit model. This estimator approximates the dynamic logit model based on the QE model, and the approximate model allows a simple sufficient statistic for each intercept. The parameters are interpreted the same way as in the actual DL model.

We obtained the approximate model by log-probability linearization of the DL model defined by the above equation, namely:


$$logp\left(y_{i}|\alpha_{i},X_{i},y_{i0}\right)=\alpha_{i}y_{i+}+\sum_{t}y_{it}x_{it}^{T}\beta +y_{i*}\gamma -\sum_{t}log\left[1+exp\left(\alpha_{i}+x_{it}^{T}\beta +y_{i,t-1}\gamma \right)\right]$$ (16)

Nonlinear component approximation for the first-order Taylor series expansion, $\alpha_{i}=\overset{―}{\alpha }$, $\beta =\overset{―}{\beta }$, γ=0:


$$\sum_{t}log\left[1+exp\left(\alpha_{i}+x_{it}^{T}\beta +y_{i,t-1}\gamma \right)\right]\approx \sum_{t}\{log\left[1+exp\left({\overset{―}{\alpha }}_{i}+x_{it}^{T}\overset{―}{\beta }\right)\right]+{\overset{―}{q}}_{it}\left[\alpha_{i}-{\overset{―}{\alpha }}_{i}+x_{it}^{T}\left(\beta -\overset{―}{\beta }\right)\right]\}+{\overset{―}{q}}_{i1}y_{i0}\gamma +\sum_{t>1}{\overset{―}{q}}_{it}y_{i,t-1}\gamma$$ (17)

The ${\overset{―}{q}}_{it}=\frac{exp\left({\overset{―}{\alpha }}_{i}+x_{it}^{T}\overset{―}{\beta }\right)}{\left[1+exp\left({\overset{―}{\alpha }}_{i}+x_{it}^{T}\overset{―}{\beta }\right)\right]}$.

Under this approximate model (from now on referred to as QEpseudo), the joint probability of $y_{i}$ is:


$$p\left(y_{i}|\alpha_{i},X_{i},y_{i0}\right)=\frac{exp\left(y_{i+}\alpha_{i}+\sum_{t}y_{it}x_{it}^{T}\beta -\sum_{t}{\overset{―}{q}}_{it}y_{i,t-1}\gamma +y_{i*}\gamma \right)}{\sum_{z}exp\left(z+\alpha_{i}+\sum_{t}z_{t}x_{it}^{T}\beta -\sum_{t}{\overset{―}{q}}_{it}z_{i,t-1}\gamma +z_{i*}\gamma \right)}$$ (18)

Given $\alpha_{i}$ and $X_{i}$, the above model corresponds to the quadratic, exponential model [30], where the second-order interaction is equal to γ when a continuous response variable is involved and 0 otherwise.

Under the approximate model, each $y_{i+}$ is a sufficient statistic for the associated parameter $\alpha_{i}$. By conditioning the total score, the joint probability of $y_{i}$ becomes:


$$p\left(y_{i}|X_{i},y_{i0},y_{i+}\right)=\frac{exp\left(\sum_{t}y_{it}x_{it}^{T}\beta -\sum_{t}{\overset{―}{q}}_{it}y_{i,t-1}\gamma +y_{i*}\gamma \right)}{\sum_{z:z_{+}=y_{i+}}exp\left(\sum_{t}z_{t}x_{it}^{T}\beta -\sum_{t}{\overset{―}{q}}_{it}z_{i,t-1}\gamma +z_{i*}\gamma \right)}$$ (19)

Where the individual intercept $\alpha_{i}$is eliminated.

In 2012, Bartolucci and Nigro proposed a pseudo-CML estimator based on the ap-proximate model described in the above formula. The estimator is based on the following two-step procedure:

(1) A preliminary estimate of regression parameter $\tilde{\beta }$ is the condition of the static Logit model by maximizing the logarithmic likelihood to calculate. In addition, the probability ${\overset{―}{q}}_{it}$ is in $\overset{―}{\beta }=\hat{\beta }$, and ${\overset{―}{\alpha }}_{i}$ is equal to the static logit model calculated under the condition of maximum likelihood estimate.
(2) The parameter vector $\theta ={\left(\beta^{T},\gamma \right)}^{T}$ is estimated by maximizing the conditional logarithmic likelihood:

$${𝓁}_{p}\left(\theta |\overset{―}{\beta }\right)=\sum_{i}I\left\{0<y_{i+}<T\right\}logp_{p}\left(y_{i}|X_{i},y_{i0},y_{i+}\right)$$ (20)

A simple Newton-Raphson algorithm can achieve the maximum value of ${𝓁}_{p}\left(\theta |\overset{―}{\beta }\right)$ to obtain the pseudo-CML estimator ${\hat{\theta }}_{p}={\left({\hat{\beta }}_{p}^{T},{\hat{\gamma }}_{p}\right)}^{T}$for the structural parameters of the DL model.

CML estimation is practical for applications based on small-*T* panel data and highly unbalanced long panel data. However, CML estimation requires the maximum singular likelihood function. When *T* is large, calculating the singular likelihood function with the standard method means an excessive burden, thus limiting the practicality of this technique. It may become unavailable in some cases. One way to overcome this calculation problem is to use the recursive algorithm proposed by Bartolucci et al. (Bartolucci et al., 2021) [48]. The recursive algorithm avoids the computational burden of the QE model on large-T data sets, which broadens the applicability of conditional reasoning in dynamic models. Therefore, we can adapt the dynamic logit model in this paper.

### Empirical analysis

#### Survival analysis results.

**Fig 4:**
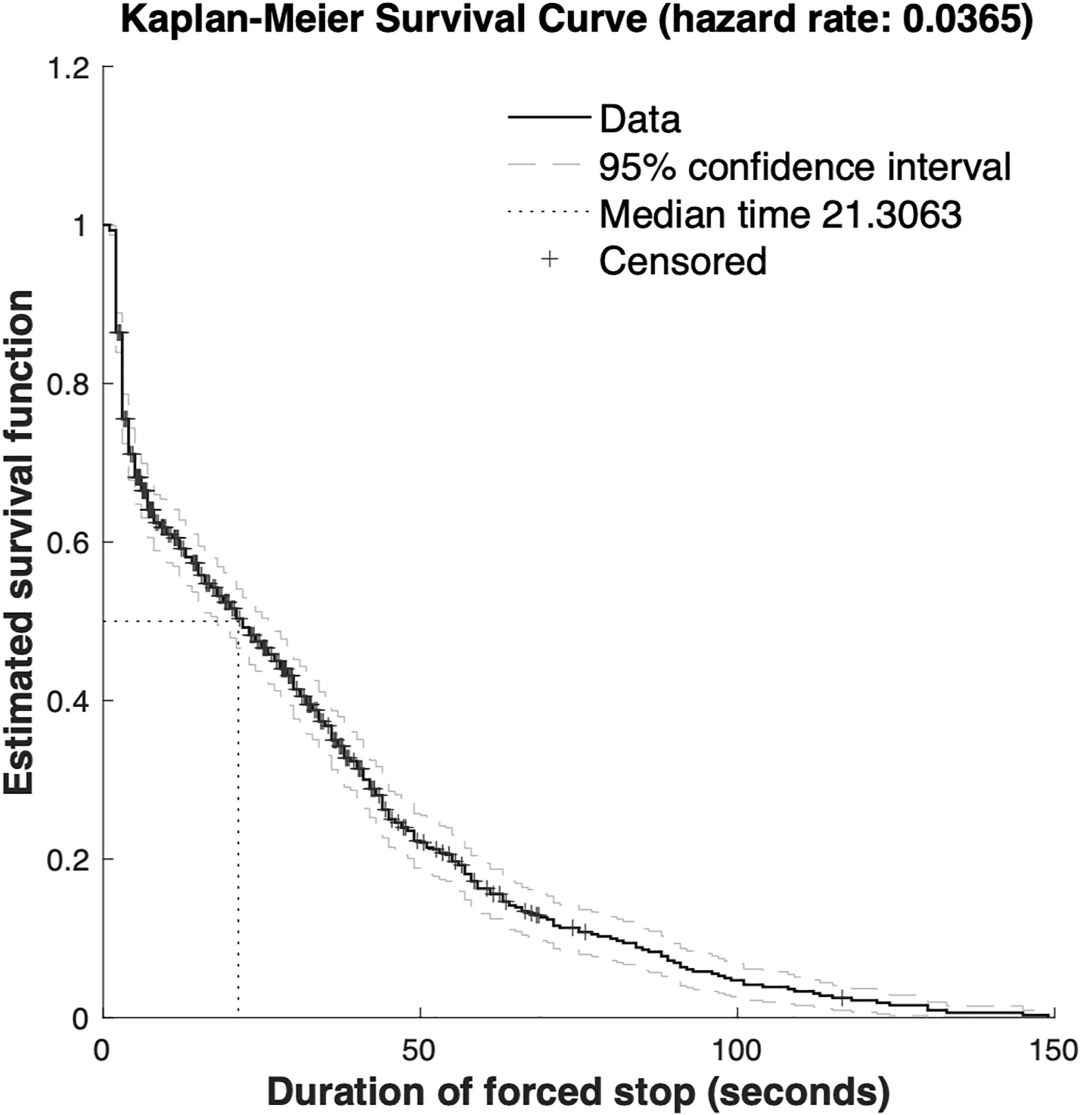
Kaplan-Meier Survival Curve of Forced Stop Duration.

The overall survival of forced parking duration was analyzed using the Kaplan–Meier method, and the resulting survival function is shown in Fig 4 (x-axis in seconds). The analysis is based on a sample of 879 forced stop events. The survival probability—i.e., the likelihood that a vehicle continues to wait without dropping off—declines as the forced stop duration increases. The median survival time is approximately 21.3 seconds, and the average forced stop duration is 27.08 seconds. The survival probability falls to near zero by around 140 seconds, indicating that the majority of vehicles choose to drop off passengers before reaching this duration threshold.

#### 1 ) Univariate analysis.

**Fig 5:**
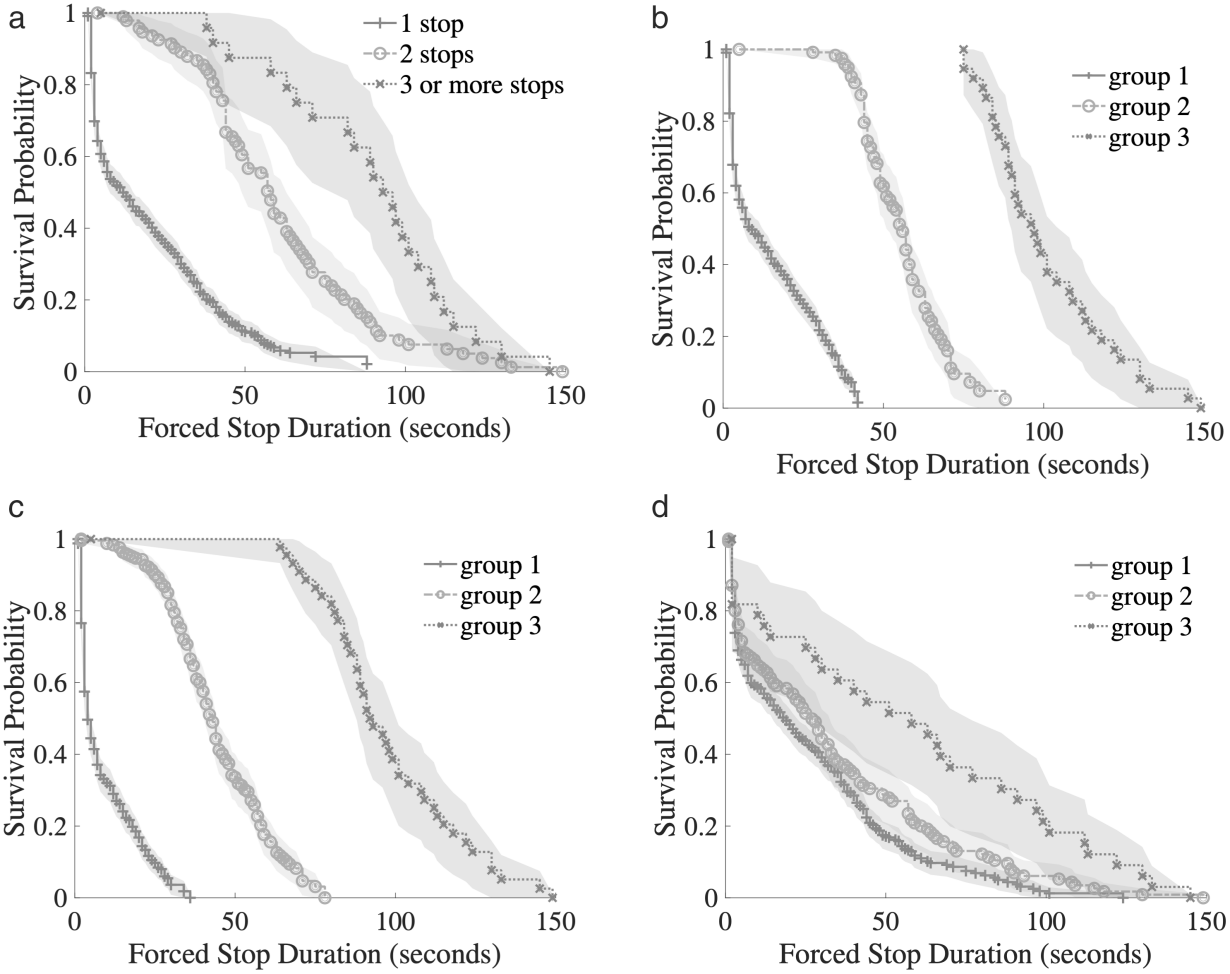
Kaplan-Meier Survival Curve of four 3-Categorical Variables: (a) Survival Curve by Forced Stop Count Groups; (b) Survival Curve by Cumulative Forced Stop Duration Groups; (c) Survival Curve by Travel Time Groups; (d) Survival Curve by P_drop-off_ Groups.

To test whether categorical variables in drop-off behavior influence forced parking duration, the Kaplan–Meier model was applied for univariate analysis. Fig 5 presents the survival curves for four variables: (a) number of forced stops, (b) cumulative forced stop duration, (c) travel time, and (d) drop-off probability at the current location. Each variable is divided into three categorical groups for comparative purposes; however, the grouping method varies by variable.

In Fig 5a, the grouping is based on the actual count of forced stops: one, two, or three-or-more stops. In contrast, Figs 5b–5d involve clustering continuous variables into three representative behavioral groups (low, medium, and high levels) using unsupervised methods such as K-means. As a result, while all subplots contain three curves, the basis for classification differs, which partly explains the variation in survival trends across subplots. Specifically, the survival curves in Fig 5(a) show that a higher number of forced stops significantly increases parking duration. Vehicles that experienced only one stop had an average waiting time of 22.13 seconds, while those with two stops waited 61.39 seconds, and vehicles with three or more stops waited on average 89.88 seconds. This trend may be shaped not only by external factors (e.g., traffic congestion and infrastructure layout), but also by behavioral elements—particularly the sunk cost effect. That is, drivers may be psychologically inclined to continue waiting after experiencing multiple delays, reasoning that since time has already been invested, continuing to wait may seem justifiable even when suboptimal. Meanwhile, in Figs 5b–5d, the observed trends reflect different behavioral clusters of cumulative duration, travel time, and drop-off probability. These clusters reveal how different patterns of exposure or experience influence the duration of continued waiting, and further validate the heterogeneity in driver responses during forced stops.

**Fig 6:**
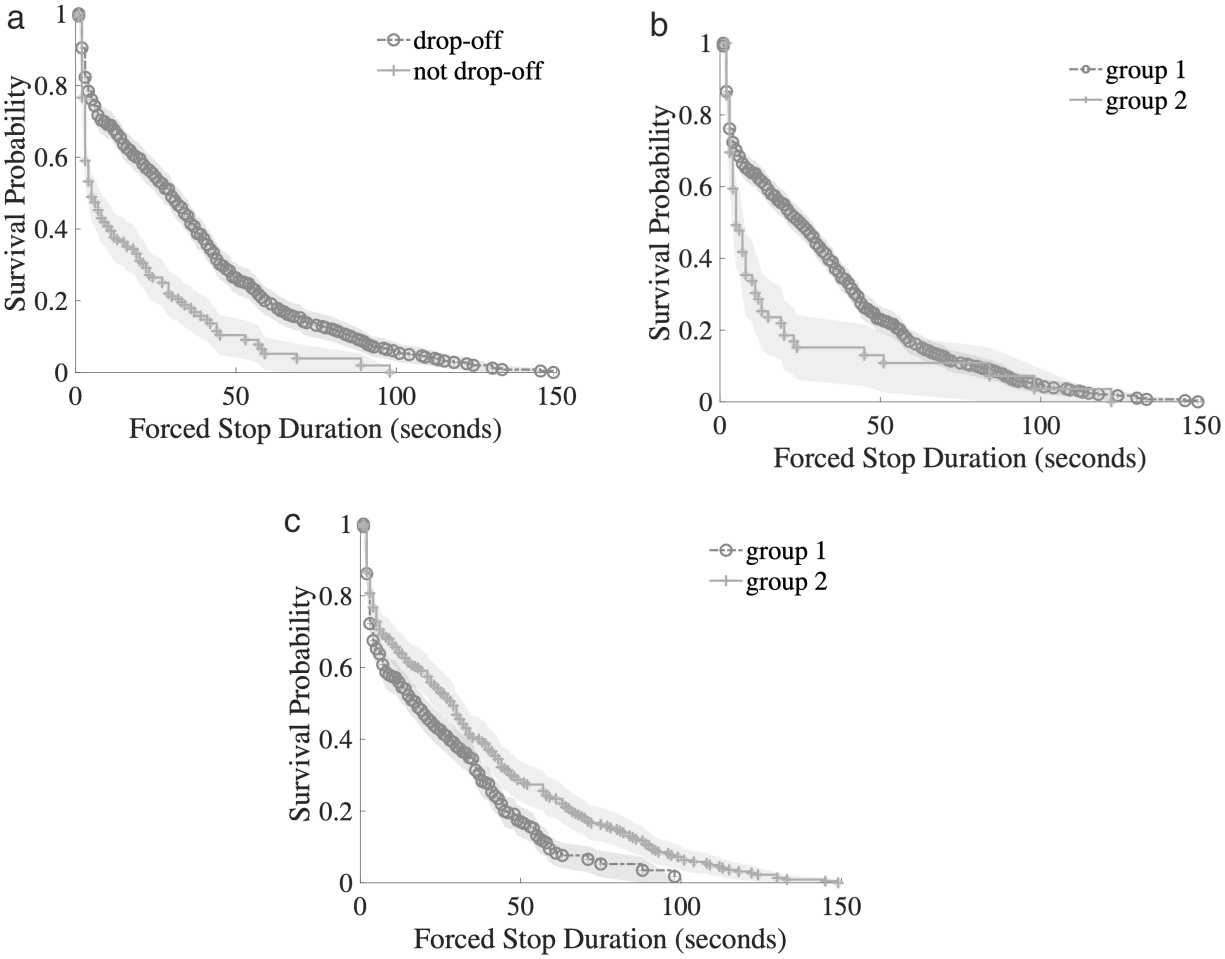
Kaplan-Meier Survival Curve of three 2-Categorical Variables: (a) Survival Curve by Preceding Vehicle Drop-off Status Groups; (b) Survival Curve by Drop-off Duration Groups; (c) Survival Curve by Forced Stop Location Groups.

In Fig 6, (a) ~ (c) represent the survival curves of the group of preceding vehicle drop-off status, drop-off duration and forced stop location, respectively. In the survival function curve of whether the front vehicle drops off or not (Fig 5(a)), the blue curve (drop-off, the front vehicle drops off) has a slower decline and a higher survival probability, indicating that the parking duration is longer; the green curve (not drop-off, the front vehicle does not drop off) has a faster decline and a lower survival probability, indicating that the parking duration is shorter. This result shows that when the front vehicle drops off, the parking duration is shorter. Forced stops last longer; When the car is not unloaded, the duration of forced parking is shorter. In a forced parking situation, the vehicle behind will take longer to stop, not only because of the physical traffic block, but also because of the Sunk Cost Effect. When the vehicle behind is forced to stop due to the vehicle in front of it, the driver may have to wait for a certain period. And choose to continue to wait for a longer time, rather than voluntarily drop off guests. After experiencing a certain amount of waiting time, the driver will tend to think that “since you have waited, it is better to wait”, rather than based on the optimal decision (following the previous car to drop off passengers). To reduce the impact of drop-off on subsequent vehicles, a special drop-off area is set up so that drop-off vehicles will not directly affect the traffic flow of the carriageway and reduce the probability of forced parking of rear vehicles. The Log-rank test of p < 0.005 for all variables in Fig 5 and Fig 6 shows that the above variables have a statistically significant effect on the forced stop duration.

#### 2 ) Multifactor cox regression.

Based on the univariate analysis, significant variables and cluster labels were selected as covariates to construct the Cox proportional hazards model and evaluate the dynamic influence of each factor on the duration of forced parking. The regression results show that the cumulative duration of forced stop, the duration of passenger drop-off, the location of forced parking of the vehicle, travel time and the probability of passenger drop-off during the current forced parking time are significant risk factors (P ≤ 0.02). Among them, the hazard ratios of drop-off duration, forced parking location, and the probability of drop-off during the current forced parking time are greater than 1, indicating that their increase will accelerate the occurrence of drop-off events. The risk ratio of cumulative forced parking time to travel time is less than 1, indicating that the larger this value is, the more inclined the driver is to extend the waiting time. The model estimation results are shown in Table 1 as follows.

**Table 1:** Estimation Results of the Cox Proportional Hazards Model.

Concomitant variable	B	Exp(B)	SE	P	95% CI(B)	
					Lower	Upper
$t_{cum}$	-2.709	0.067	0.221	0.000	0.043	0.103
$t_{drop}$	0.323	1.382	0.139	0.020	1.052	1.814
$L_{i}$	0.851	2.342	0.092	0.000	1.956	2.805
$t_{travel}$	-2.315	0.099	0.161	0.000	0.072	0.135
$P_{patience}$	3.273	26.4	0.247	0.000	16.262	42.860

### Discrete choice model results

#### 1 ) Estimated result.

The random effect static logit model, fixed effect static logit model, and dynamic logit model were respectively estimated. Table 2 shows the estimation results of the decision models of the first forced parking and more than two forced parking and drop-off.

**Table 2:** Estimation Results of Each Forced Parking and Drop-off Decision Model.

Model	Var.	Random effects model			Fixed effects model			Dynamic logit model		
		Coef.	S.E.	p-value	Coef.	S.E.	p-value	Coef.	S.E.	p-value
**1**^**st**^ **forced stops**	y_it-1_	–	–	–	–	–	–	13.017	5.155	0.000
	X_1_	39.749	1.227	0.000	41.324	2.532	0.000	56.036	5.364	0.000
	X_2_	10.754	1.323	0.000	–	–	–	–	–	–
	X_3_	13.849	1.042	0.000	–	–	–	–	–	–
	X_4_	−8.518	1.476	0.000	−7.895	2.615	0.003	−18.07	4.585	0.000
	Cons.	−19.720	1.218	0.000	–	–	–	–	–	–
**2**^**nd**^**-4**^**th**^ **forced stops**	y_it-1_	–	–	–	–	–	–	1.218	0.840	0.042
	X_1_	117.711	6.079	0.000	53.258	7.926	0.000	51.958	3.161	0.000
	X_2_	55.411	12.913	0.000	–	–	–	–	–	–
	X_3_	36.766	12.623	0.004	–	–	–	–	–	–
	X_4_	−22.443	5.651	0.000	−5.479	2.161	0.011	−19.45	2.997	0.000
	X_5_	−18.921	1.785	0.000	–	–	–	–	–	–
	Cons.	−49.442	8.511	0.000	–	–	–	–	–	–

The estimation results show that the random effect model of the first forced parking drop-off decision has one more variable than the fixed effect model: The number of forced stops hurts the drop-off decision. that is, the more times of forced stop, the lower the drop-off probability because it takes longer to make a few stops in the back of a vehicle than in the front. This side illustrates the sunk cost fallacy. The higher the cost, the stronger the sunk cost effect. “The probability that the current parking time is wait patience” significantly influences whether the vehicle drops off. The positive influence indicates that the closer the waiting time is to wait patience, the higher the probability of the car dropping off. Another factor affecting the drop-off decision is the proportion of vehicle driving time (i.e., the ratio of vehicle driving time to vehicle travel time), which affects the drop-off decision negatively. In other words, the smaller the driving time ratio, the longer the cumulative parking time and the higher the probability of dropping off passengers. In the random effects model, the variables “relative parking position of the vehicle” and “probability that the current parking position is the expected parking space” positively affect the drop-off decision.

#### 2) Model comparison and verification.

To evaluate the performance of the developed models in predicting the drop-off decisions under different forced parking conditions, both in-sample and out-of-sample tests using the available dataset were conducted. The models tested include:

- REM: Random Effects Logit Model
- FEM: Fixed Effects Logit Model
- DLM: Dynamic Logit Model
- COX: Cox Proportional Hazards Model

The prediction performance was assessed using the ROC (Receiver Operating Characteristic) curve and the area under the ROC curve (AUC), a widely used evaluation metric for binary classification models.

#### (1) In-sample Test.

To evaluate the predictive performance of the proposed models, we categorize vehicles based on the number of forced stops they experience before dropping off. This is consistent with the system structure illustrated in Fig 1. Specifically:

- The term “first forced stops” refers to vehicles that make drop-off decisions at the first instance of forced parking.
- The term “second and more forced stops” applies to vehicles that do not drop off at the first opportunity and undergo two or more forced stops before deciding to drop off.

These definitions form the grouping criteria for the analysis results presented in Tables 3, which report the model performance for different categories of forced stop events.

**Table 3:** ROC Results Predicted in the Sample.

Number of stops	Model^1^	ROC area	Std. err.	95% conf. interval
**All**	COX	0.7151	0.0210	[0.67422 0.75781]
**The first forced stops**	REM	0.8198	0.0033	[0.81433 0.82526]
	FEM	0.8222	0.0033	[0.81665 0.82752]
	DLM	0.8857	0.0029	[0.88109 0.89022]
**The second and more forced stops**	REM	0.8393	0.0120	[0.81936 0.85740]
	FEM	0.7358	0.0148	[0.71282 0.75830]
	DLM	0.7460	0.0102	[0.72992 0.76235]

1 COX is a Cox Proportional Hazards Model, REM is a random effects model, FEM is a fixed effects model, and DLM is a dynamic Logit model.

**Fig 7:**
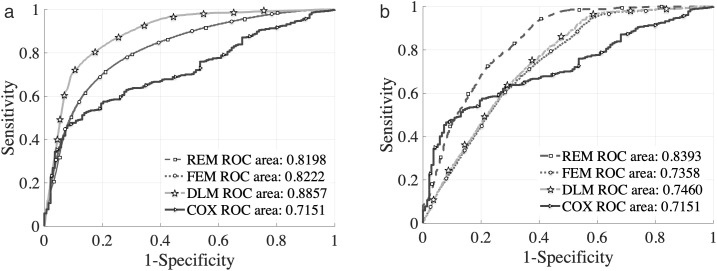
ROC for Classification of Test Results: (a) ROC Curve of the First Forced Drop-off Decision Models; (b) ROC Curve of the Decision Models for Second and More Forced Parking and Drop-off.

Fig 7 and Table 3 present the ROC results obtained from using the models to simulate the training (in-sample) dataset. In the ROC curve, the horizontal axis represents 1-specificity (i.e., false positive rate), and the vertical axis represents sensitivity (i.e., true positive rate), which together describe the classification trade-off of the model at different threshold settings. The AUC (Area Under the Curve) values indicate that all models achieve an overall prediction accuracy above 70%. For the first forced stop decision, the dynamic logit model (DLM) achieves the highest AUC (88.57), showing superior predictive ability. The random effect model (REM) also performs consistently well, especially in the case of second or subsequent forced stops. The results suggest that while the dynamic model (DLM) offers the best prediction accuracy when sufficient behavioral data is available (i.e., the first forced stop dataset, which contains a larger number of observations), the REM exhibits greater stability and robustness across different scenarios.

#### (2) Out-of-sample Test.

**Fig 8:**
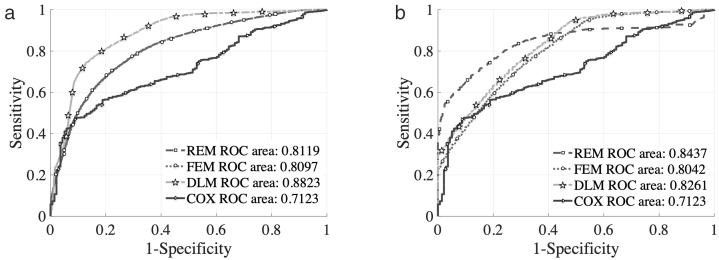
Out-of-sample Test Results of the Drop-off Decision Models: (a) ROC Curve of the First Forced Drop-off Decision Models; (b) ROC Curve for Second and More Forced Parking and Drop-off.

To verify the model’s generalizability, data from adjacent time periods on the same day were used for prediction. As shown in Fig 8 and Table 4, all models again demonstrate good predictive performance, with AUC values above 70%. The DLM continues to perform best in predicting first forced stop behavior, while REM remains the most robust model across scenarios.

**Table 4:** ROC Results Predicted for Out of the Sample.

Number of stops	Model^2^	ROC area	Std. err.	95% conf. interval
**All**	COX	0.7123	0.0210	[0.66954 0.75321]
**The first forced stops**	REM	0.8119	0.0056	[0.80251 0.82107]
	FEM	0.8097	0.0056	[0.80030 0.81894]
	DLM	0.8823	0.0050	[0.87774 0.89356]
**The second and more forced stops**	REM	0.8437	0.0160	[0.84240 0.85212]
	FEM	0.8042	0.0120	[0.80141 0.81763]
	DLM	0.8261	0.0150	[0.81528 0.83443]

2 COX is a Cox Proportional Hazards Model, REM is a random effects model, FEM is a fixed effects model, and DLM is a dynamic Logit model.

In summary, all model types are effective in modelling drop-off decisions, with the DLM offering the highest predictive accuracy in the case of the first forced stop, while REM demonstrates strong generalization performance and consistency across both stages of decision-making. Compared with the Static Logit Model, the Dynamic Logit Model (DLM) better captures the sequential behavioral patterns of drivers, as it incorporates time-dependent variables and historical decision information. This enables the DLM to reflect cumulative effects and learning behavior over repeated forced stops, improving prediction when sample size is sufficient. On the other hand, the Static Logit Model, while structurally simpler and easier to estimate, is more robust under small-sample conditions. It offers stable performance with fewer parameters, making it suitable for scenarios where driver behavior does not evolve significantly over time. The complementary strengths of both models suggest that their applicability depends on the availability of behavioral data and the temporal characteristics of the decision process.

## Conclusion

This study aims at the operational characteristics of taxi drop-off lanes in large-scale comprehensive passenger transport hubs and constructs a first-in-first-out taxi drop-off decision-making model, covering both static and dynamic Logit models. Both are based on panel data and consider the influence of different vehicles, time and space on drop-off decisions. The model assumes that in a forced parking situation, taxis in the fleet decide whether to let passengers get off based on factors such as the distribution of passengers’ patience, parking positions, expected parking Spaces, and driving time. The distribution of passenger patience is set based on actual data. The main explanatory variables extracted include “the probability that the current forced parking time reaches’ waiting patience ‘”,” the relative parking position of the vehicle “, “the probability that the current parking position is the expected parking space”, and “the proportion of driving time”, simplifying the model while retaining the original index information as much as possible.

The analysis results show that “the probability of the current forced parking time reaching ‘waiting patience’” plays a decisive role in the decision to get off passengers. The model validation results indicate that both static and dynamic Logit models, as well as the Cox proportional hazards model, can effectively capture the drop-off decision process, with all achieving prediction accuracies above 70%. In particular, when sufficient behavioral data are available – such as in first forced stops – the dynamic Logit model shows the highest prediction accuracy (AUC = 88.57%), while the random effects static Logit model demonstrates more stable performance across varying data sizes.

To further reveal the temporal dynamic of drop-off behavior, this study introduces survival analysis methods – especially the Cox proportional hazards model – to supplement the temporal aspects that the static models fail to capture. The Survival analysis results confirm that cumulative forced parking time, travel time, and the number of forced stops are all significantly associated with longer parking durations, which in turn reduce the probability of choosing to drop-off. This is consistent with the behavioral interpretation based on the sunk cost effect: drivers who have already invested considerable time in waiting are more likely to continue waiting rather than take immediate action to drop off passengers.

Figs 4-7 and Tables 1-4 provide robust empirical support for these findings. Together, they validate the proposed modeling framework’s ability to simulate and predict taxi behavior in congested drop-off lanes. Compared to previous studies that relied primarily on static models or proximity-based heuristics, this research introduces a dual modeling approach that captures both the time-dependent nature of decision-making and cumulative behavioral effects. The hybrid use of survival analysis and discrete choice modeling provides a more comprehensive behavioral perspective on taxi operations in FIFO lanes. Its application to Nanjing South Railway Station demonstrates strong real-world relevance and policy implications..

Based on these insights, the following management implications are proposed, combining predictive insights with behavioral mechanisms and spatial value considerations:

**Set up a “no-waiting area”:** Designate a specific zone within the drop-off lane where taxis are required to drop off passengers immediately upon stopping. While this may slightly increase walking distance for some passengers, it greatly reduces idle waiting and improves turnover. The implementation cost is relatively low, requiring mainly traffic markings and signage, and it enhances spatial efficiency in high-demand areas.

**Implement parking pricing for prolonged stops:** Given that transport hubs such as airports and high-speed rail stations are premium spatial locations, we propose introducing tiered or time-dependent pricing policies for taxis that exceed a certain waiting threshold. For instance, parking beyond 60 seconds may incur progressive fees, which serves both as a behavioral nudge and a mechanism to internalize the opportunity cost of occupying scarce space. This complements the findings from our survival and hazard models, where longer stops correlate with inefficiency and sunk cost effects. Although it introduces moderate system setup and enforcement costs, this pricing policy can generate revenue while improving operational discipline.

**Set a mandatory drop-off time threshold:** Based on the median waiting time from the survival curve (e.g., 21.3s), a hard time limit can be established beyond which vehicles must complete drop-off. This measure is low-cost and can be enforced via on-site staff or automated reminders..

**Deploy real-time guidance and alert systems:** Utilize Variable Message Signs (VMS), voice prompts, or AI-enhanced cameras to provide in-situ feedback to drivers—especially those with high cumulative forced stop durations. While initial investment is higher, this infrastructure enhances responsiveness, supports data-driven operations, and can integrate with broader intelligent transportation systems (ITS).

Together, these measures balance cost-effectiveness and behavioral incentives while acknowledging the strategic importance of limited space in high-density transport hubs. The modeling framework developed in this study offers empirical support for the design and evaluation of traffic management strategies, particularly in operational environments where lane turnover and drop-off efficiency are critical.

Future research may extend this work by incorporating more flexible lane configurations (e.g., non-FIFO systems), integrating real-time data for adaptive prediction (e.g., through reinforcement learning), validating behavioral assumptions via stated preference surveys or experiments, and expanding the scope to consider interactions between multiple transport modes such as ride-hailing and private cars. These directions will help build more comprehensive and resilient drop-off systems in complex multimodal environments..
